# Tailoring Interfacial Activity of pH-Driven Shellac–Chitosan Nanocomposites via Solution Addition Sequence for Pickering Emulsion Stabilization

**DOI:** 10.3390/foods14203556

**Published:** 2025-10-18

**Authors:** Yi Yuan, Luping Qu, Tingyong Zheng, Tangyu Yang, Huan Liu, Yajun Li, Shutao Liu

**Affiliations:** 1 College of Biological Science and Engineering, Fuzhou University, Fuzhou 350108, China; 2 School of Food Science and Technology, Dalian Polytechnic University, Dalian 116034, China; 3 Department of Biotechnology, Faculty of Bioscience Engineering, Ghent University, Coupure Links 653 B, 9000 Ghent, Belgium

**Keywords:** shellac, chitosan, nanocomposites, pH-driven, interfacial activity

## Abstract

The pH shift generated by mixing alkaline shellac (SH) and acidic chitosan (CS) solutions may drive the formation of nanocomposites with interfacial activity. However, how the solution addition sequence affects their formation and properties remains unclear. In this study, we systematically investigated the influence of addition order on the formation, physicochemical properties, and interfacial activity of SH-CS nanocomposites. The results showed that pH variation during mixing promoted nanocomposite formation, with optimal electrostatic interactions occurring at a final pH near 5.0. The most efficient assembly was achieved at an SH: CS mass ratio of 2:3. FTIR and dissociation experiments confirmed that hydrogen bonding, hydrophobic effects, and electrostatic interactions jointly governed the assembly process. Importantly, the addition sequence determined the nanocomposite structure: adding SH to CS produced core–shell structures, whereas the reverse order yielded co-assembled hybrid nanocomposites. These distinct structures directly impacted interfacial behavior. The co-assembled nanocomposites effectively balanced the inherent hydrophobicity of SH and hydrophilicity of CS, achieving moderate wettability. This balance significantly reduced interfacial tension, thereby enhancing emulsifying performance. Overall, this study underscores the critical role of addition sequence in tailoring the properties of pH-driven SH-CS nanocomposites and highlights their strong potential as high-performance Pickering emulsifiers.

## 1. Introduction

Pickering emulsions are stabilized by solid or colloidal particles that irreversibly adsorb at the oil-water interface, forming a protective layer that increases steric hindrance and reduces interfacial fluidity [1,2]. Compared with conventional emulsions, they exhibit superior stability by resisting coalescence and Ostwald ripening, thereby preventing phase separation caused by agglomeration or flocculation [3]. Owing to these advantages, Pickering emulsions are widely applied in the chemical, pharmaceutical, and food industries. In particular, food-grade Pickering emulsions have been utilized for encapsulating and delivering bioactive compounds, fortifying meat products, and developing low-fat foods [4,5,6]. However, most conventional Pickering stabilizers are inorganic particles or synthetic polymers, which are unsuitable for food applications. Consequently, food-grade stabilizers derived from natural proteins and polysaccharides have attracted increasing interest [7]. Despite their potential, proteins are often pH-sensitive and thermally unstable, while polysaccharides are excessively hydrophilic—both of which limit their effectiveness as stabilizers [8,9]. Therefore, developing novel food-grade particles or strategies to enhance the functionality of existing ones remains an urgent task.

Shellac (SH) is a natural resin secreted by lac insects. It is generally recognized as safe (GRAS) by the U.S. Food and Drug Administration (FDA) and approved as a food additive (E904) by the European Food Safety Authority (EFSA) [2]. Structurally, SH is a polyester composed of aleuritic acid and cyclic terpene acid. It is soluble in ethanol but insoluble in pure water due to its multipolar molecular structure. In addition, unesterified carboxyl groups impart pH-dependent solubility: SH dissolves in alkaline solutions but precipitates in acidic media. These specific solubilities enable the preparation of SH nanoparticles through anti-solvent or pH-driven precipitation [10,11]. Owing to its amphiphilic molecular structure, SH nanoparticles theoretically possess potential as Pickering stabilizers. However, its pronounced hydrophobicity often hinders interfacial adsorption. For instance, SH nanoparticles formed by anti-solvent precipitation can stabilize Pickering emulsions but display poor storage stability, showing phase separation and demulsification over time [12]. Enhancing the hydrophilicity of SH nanoparticles is therefore critical for improving their interfacial performance. Previous studies have explored hybrid particles such as SH-calcium alginate dimer particles, where SH particles provide the hydrophobic domain and calcium alginate microgels supply hydrophilic functionality, effectively stabilizing water-in-oil or oil-in-water Pickering emulsions depending on particle size [13]. Other approaches combined SH with polylactic acid and polyethylene glycol modification, converting SH-based particles into hydrophilic moieties [14]. Alternatively, grafting hydrophilic polymers onto SH nanoparticles or constructing SH-based nanocomposites has been shown to enhance emulsification efficiency [15,16,17].

Chitosan (CS) is a linear cationic polysaccharide composed of N-acetylglucosamine and D-glucosamine units linked by β(1→4) bonds, derived from the deacetylation of chitin—the second most abundant polysaccharide in nature. With a pKa of 6.2–7.0, CS is soluble in acidic conditions, where protonated amine groups confer a positive charge [18]. However, its strong hydrophilicity under acidic environments limits its ability to stabilize emulsions, particularly for highly deacetylated CS [19]. To overcome this drawback, CS has been combined with hydrophobic molecules to form complexes with improved emulsifying ability. For example, CS-phytosterol complexes formed via electrostatic interactions improve emulsion stability [20], and a CS-to-zein ratio of 1:20 yields nearly neutral wettability (contact angle ≈ 90°), promoting emulsion construction [21]. Furthermore, interactions between CS and SH nanoparticles prepared by anti-solvent precipitation have been reported to improve emulsifying properties [16]. Importantly, SH and CS exhibit complementary solubility and charge characteristics: SH dissolves under alkaline conditions and carries a negative charge, while CS dissolves under acidic conditions and carries a positive charge [10,18]. This complementarity makes the direct construction of SH-CS nanocomposites via simple mixing both feasible and attractive, as it balances hydrophilic and hydrophobic properties, thereby broadening their potential in food emulsions. Nevertheless, the influence of the solution addition sequence on SH–CS self-/co-assembly behavior and the resulting interfacial activity has not been quantitatively examined. Moreover, the dynamic pH trajectory and variations in charge strength during mixing—key factors likely govern assembly structure—have not been systematically analyzed. Addressing these knowledge gaps is essential for elucidating the interfacial mechanisms underlying SH–CS nanocomposite-stabilized emulsions.

We hypothesized that local pH changes induced by different solution addition orders would trigger distinct assembly behaviors of SH and CS, leading to SH-CS nanocomposites with varied physicochemical properties and interfacial activities. Therefore, this study explored the pH-driven self- and co-assembly of SH–CS nanocomposites under different addition sequences and examined their effects on the resulting physicochemical properties and interfacial behavior. Specifically, we investigated changes in turbidity and zeta potential at different pH levels and the effects of SH: CS mass ratios on particle size, polydispersity index (PDI), and zeta potential, as well as colloidal stability. We further characterized nanocomposite morphology and analyzed the dominant interaction forces governing assembly. Finally, we systematically evaluated the interfacial activity of the nanocomposites. This work deepens the understanding of polymer self-/co-assembly mechanisms and provides a foundation for the rational design of high-performance, food-grade Pickering emulsifiers.

## 2. Materials and Methods

### 2.1. Materials

Food-grade bleached SH was obtained from Yunnan Yongde Songhualin Chemicals Co., Ltd. (Lincang, China). CS (molecular weight 150 kDa, degree of deacetylation 85%) was purchased from Shandong AK Biotech Co., Ltd. (Jinan, China). Soybean oil was obtained from Yihai Kerry Arawana Holdings Co., Ltd. (Shanghai, China) and consisted mainly of linoleic acid (approximately 50–57%), oleic acid (22–30%), palmitic acid (9–13%), and linolenic acid (5–9%). Ninhydrin, hydrochloric acid, sodium hydroxide, acetic acid, and other analytical-grade reagents were purchased from Sinopharm Chemical Reagent Co., Ltd. (Shanghai, China). Sodium dodecyl sulfate (SDS) was obtained from Xilong Scientific Co., Ltd. (Shantou, China). Deionized water was used as the solvent in all experiments.

### 2.2. Preparation of SH-CS Nanocomposites

SH was dispersed in 0.1 M sodium hydroxide and stirred overnight at 25 °C to ensure complete hydration. The pH was then adjusted to 8.0 with 4 M hydrochloric acid to obtain a 3.5 mg/mL SH solution. CS was dissolved in 1% (*v*/*v*) acetic acid and stirred overnight at 25 °C to obtain a 3.5 mg/mL CS solution. Nanocomposites with different SH:CS mass ratios (4:1, 2:1, 4:3, 1:1, 2:3, 1:3, and 1:6) were prepared by adding the SH solution into the CS solution in a 2 cm diameter glass vial under continuous stirring (550 rpm, 25 °C). The final pH was adjusted to 5.0 ± 0.05 with sodium hydroxide or hydrochloric acid. Preliminary experiments identified this pH as optimal, as it allowed shellac to form a stable dispersion of negatively charged nanoparticles without aggregation, while maintaining chitosan in its protonated form to promote electrostatic complexation. The mixture was stirred continuously for 30 min, and the resulting samples were designated as SH@CS. For the reverse process, the CS solution was added into the SH solution under identical conditions, and the resulting nanocomposites were labeled CS@SH.

### 2.3. Turbidity and Zeta Potential at Different pH Levels

SH and CS solutions were diluted tenfold, and SH@CS and CS@SH dispersions (SH:CS = 2:3) were prepared as described in Section 2.2. The pH of each dispersion was adjusted from 1.0 to 6.0. Turbidity and zeta potential were measured to evaluate structural transitions and molecular interactions as a function of pH. For turbidity, pH intervals of 0.5 were used, while for zeta potential, intervals of 1.0 were applied. Turbidity was determined by measuring absorbance at 600 nm using a UV-Vis spectrophotometer (GENESYS 10S, Thermo Fisher Scientific Inc., Waltham, MA, USA) in a 1 cm × 1 cm pathlength plastic cuvette at 25 °C. Based on the turbidity-pH curves, characteristic pH values were identified following Wei et al. [22]: pH_c_, the critical pH at which turbidity begins to increase (formation of soluble complexes); pH_φ1_, the pH corresponding to the onset of insoluble complex formation (sharp turbidity rise); pH_opt_, the pH of maximum turbidity; and pH_φ2_, the pH where complexes dissociate upon further acidification. Zeta potential was measured using a Nano-ZS laser light scattering instrument (MPT-2, Malvern Instruments Ltd., Malvern, Worcestershire, UK) at 25 °C. Each sample was analyzed in triplicate.

### 2.4. Effect of Composition Ratio on Nanocomposite Properties

#### 2.4.1. Turbidity

Turbidity of nanocomposites prepared at different SH:CS ratios was determined as described in Section 2.3.

#### 2.4.2. Particle Size, PDI, and Zeta Potential

Particle size, PDI, and zeta potential were determined using a Nano-ZS laser light scattering instrument (MPT-2, Malvern 194 Instruments Ltd., Malvern, Worcestershire, UK). For particle size and PDI measurements, samples were diluted 100-fold to minimize multiple scattering. Measurements were conducted at 25 °C with a backscattering angle of 175°. Equilibration time and dispersant viscosity were automatically set by the instrument software. Each sample was tested in triplicate. Zeta potential was determined as described in Section 2.3.

#### 2.4.3. Dispersed Fraction After Storage

The dispersed fraction (DF) after storage—also referred to as assembly rate in previous studies—was determined following a reported method with minor modifications [23]. Freshly prepared dispersions were stored at 4 °C for 15 days. Precipitates were collected by centrifugation at 7000 rpm for 5 min, dried, and weighed. DF (%) was calculated using Equation (1).

(1) $$DF\;\left(\%\right)=\frac{W_{t}-W_{p}}{W_{t}}\times 100$$

where *W_t_* (mg) is the total mass of SH and CS in the dispersion, and *W_p_* (mg) is the mass of the washed precipitate dried to constant weight.

### 2.5. Transmission Electron Microscopy (TEM)

Nanocomposite morphology was examined using TEM (Tecnai G2 F20, FEI, Hillsboro, OR, USA) following Geng et al. [24]. Samples were diluted 20-fold, and a drop of suspension was placed on a carbon-coated 200-mesh copper grid and air-dried at 25 °C. No staining was applied, as the intrinsic electron density of SH and CS provided sufficient contrast.

### 2.6. Fourier Transform Infrared Spectroscopy (FTIR)

FTIR analysis was performed following the method of Yuan et al. [25] with minor modifications. Samples were freeze-dried for 48 h and stored in a desiccator before use to minimize water absorption. The freeze-dried nanocomposites were mixed with KBr (1:99, *w*/*w*), ground thoroughly, and pressed into transparent pellets at 10 MPa. FTIR spectra were collected using a Thermo Nicolette 6700 spectrophotometer (Thermo Fisher Scientific, Waltham, MA, USA) over the range 400–4000 cm^−1^ at 4 cm^−1^ resolution. Each spectrum represented the average of 32 scans. Pure SH nanoparticle (pH 5) and freeze-dried CS were used as controls.

### 2.7. Dissociation Experiment

Dissociation experiments were conducted to identify the main intra- and intermolecular interactions responsible for nanocomposite formation, following a previously described method with slight modifications [26]. The principle is that a selective dissociating agent disrupts specific intermolecular forces, causing disassembly detectable by a decrease in turbidity. Sodium chloride (NaCl) was used to probe electrostatic interactions by shielding polymer charges, while the anionic SDS was used to probe hydrophobic interactions. Specifically, 5 mL of 20 mM NaCl or SDS solution was mixed with 5 mL of nanocomposite dispersion, vortexed briefly, and the turbidity change was recorded as absorbance at 600 nm using a UV-Vis spectrophotometer (GENESYS 10S, Thermo Fisher Scientific Inc., Waltham, MA, USA). Because SDS can interact directly with cationic CS, a CS–SDS control was also prepared to correct for its intrinsic turbidity contribution.

### 2.8. Interfacial Activity

#### 2.8.1. Three-Phase Contact Angle

Three-phase contact angles were measured using an optical contact angle meter (OCA 20, DataPhysics Instruments GmbH, Filderstadt, Germany) following the method of [27] with slight modifications. Freeze-dried nanocomposites with different SH: CS ratios were compressed into disks (2.0 cm diameter, 0.1 cm thickness) at 10 MPa for 5 min. Each disk was placed in a cuvette filled with oil, and a 2 µL water droplet was gently deposited onto its surface using a syringe. Images were captured after 10 s of equilibration, and the oil-water contact angle (θ_o/w_) was calculated using the Young-Laplace equation. All measurements were conducted in triplicate.

#### 2.8.2. Interfacial Tension

Interfacial tension was determined using the pendant drop method at 25 °C with an optical contact angle meter (OCA 20, DataPhysics Instruments GmbH, Filderstadt, Germany) following [28] with minor modifications. A nanocomposite dispersion (3.5 mg/mL) was loaded into a microinjection needle inserted into a quartz glass cuvette containing purified soybean oil. A 15 µL droplet was slowly extruded, and its shape was monitored until the adsorption equilibrium (2500 s) was reached. Interfacial tension was calculated using the Young-Laplace equation.

### 2.9. Emulsification Performance

Emulsifying activity index (EAI) and emulsion stability index (ESI) were determined using turbidimetry following [29,30] with slight modifications. Specifically, equal volumes (5 mL) of nanocomposite solution and soybean oil were homogenized at 20,000 rpm for 2 min (IKA T10, IKA Works GmbH & Co. KG, Staufen, Germany), followed by ultrasonication in an ice bath using a cell disruptor with a 6 mm diameter probe (JY92-IIDN, Ningbo Scientz Biotechnology Co., Ltd., Ningbo, China). Sonication was performed at 315 W with a 2 s on/5.5 s off cycle for 11 min. Aliquots (40 µL) were collected from 0.5 cm above the vial bottom at 0 min and 30 min, respectively, then diluted with 5 mL of 0.1% SDS. Their absorbance at 500 nm was measured using a UV-Vis spectrophotometer (GENESYS 10S, Thermo Fisher Scientific Inc., Waltham, MA, USA). The EAI and ESI were calculated using Equations (2) and (3), respectively.

(2) $$EAI\;(m^{2}/g)=\frac{2\times 2.303\times A_{0}\times D_{F}}{c\times \varphi \times L\times 10^{4}}$$

(3) $$ESI\;\left(\%\right)=\frac{A_{30}}{A_{0}}\times 100$$

where *A_0_* and *A_30_* are absorbances at 0 and 30 min, respectively, *c* is the sample concentration (g/mL) in the emulsion; *D_F_* is the dilution factor, *L* is the cuvette path length (1 cm), and *φ* is the oil volume fraction, which is used to normalize the turbidity and make the EAI an expression of the interfacial area stabilized per unit mass of emulsifier, independent of the specific oil-to-water ratio used.

### 2.10. Statistical Analysis

All experiments were performed in triplicate (*n* = 3), and results are presented as mean ± standard deviation. Data were tested for normality and homogeneity of variance before analysis of variance (ANOVA). One-way ANOVA followed by Duncan’s multiple range test was performed using IBM SPSS Statistics 26. Differences were considered significant at *p* < 0.05. Graphs were generated with Origin 2021 (Professional edition).

## 3. Results and Discussion

### 3.1. Effect of pH on the Formation of Nanocomposites

**Figure 1:**
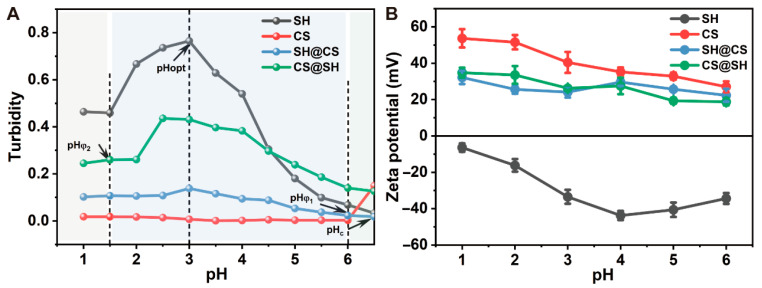
The (**A**) turbidity and (**B**) zeta potential of SH, CS, SH@CS, and CS@SH nanocomposites.

Because of their intrinsic molecular structures, SH and CS exhibit opposite solubility behaviors in aqueous media under different pH conditions. Specifically, SH dissolves in alkaline solutions, whereas CS is soluble in acidic solutions [10,18]. Variations in pH alter the molecular conformation and surface charge of both SH and CS. To elucidate the mechanism underlying nanocomposite formation driven by pH changes during SH-CS blending, it is necessary to examine their physical states and the characteristics of the resulting nanocomposites under different pH conditions. These effects were evaluated by monitoring the turbidity and zeta potential of SH, CS, and their nanocomposite dispersions (Figure 1A,B).

As shown in Figure 1A, the turbidity of CS remained nearly constant between pH 1 and 6 because of its high solubility in acidic media. With decreasing pH, protonation of amino groups along with CS molecular chains increased, leading to a progressive rise in zeta potential (Figure 1B). In contrast, SH displayed marked turbidity variations across the same pH range. At pH 6, turbidity was low because limited protonation and strong electrostatic repulsion maintained SH primarily in molecular form. As the pH decreased, enhanced protonation weakened electrostatic repulsion, promoting hydrophobic self-assembly into larger nanoparticles. At pH 3, extensive protonation of carboxyl groups further reduced interparticle repulsion, resulting in aggregation into larger clusters and a sharp rise in turbidity. Below pH 3, precipitation occurred, leading to a decrease in overall turbidity. A similar trend was reported by Lv et al. [31] when investigating pH-driven zein-SH nanoparticles for curcumin encapsulation, underscoring SH’s dominant role in precipitation behavior.

Figure 1B also indicates that SH and CS carried opposite surface charges between pH 1 and 6, supporting the potential for electrostatic interactions. Interestingly, the turbidity profiles of SH@CS and CS@SH nanocomposites differed substantially within this range. The SH@CS nanocomposite exhibited a pattern similar to that of CS but consistently displayed higher turbidity. This likely results from the rapid protonation of SH in the acidic CS environment, facilitating the rapid formation of small SH nanoparticles that subsequently interacted electrostatically with CS. The adsorption of CS onto SH nanoparticle surfaces restricted their aggregation at low pH. In contrast, the CS@SH nanocomposite showed a turbidity pattern more closely resembling that of SH. When pH > 4.5, SH@CS exhibited higher turbidity than SH alone, whereas the opposite occurred at pH < 4.5. This may be because gradual pH reduction during CS addition slowed SH self-assembly, enabling SH molecules to interact directly with positively charged CS to form nanocomposites. At pH > 4.5, SH self-assembly was minimal, and the system mainly contained free SH molecules or small particles, whereas at pH < 4.5, nanocomposite formation prevented the extensive aggregation observed in pure SH systems.

Furthermore, the zeta potentials of both nanocomposites fell between those of SH and CS, exhibiting an overall positive charge. This suggests that charge neutralization occurred during nanocomposite formation. The positively charged, high-molecular-weight CS likely masked the negative charges of SH, resulting in positively charged nanocomposites. Collectively, Figure 1A,B indicate that the optimal conditions for nanocomposite formation occurred near pH 5. At this pH, SH and CS displayed the largest potential difference (approximately 80 mV), while SH had not yet undergone extensive aggregation, allowing strong electrostatic interactions. Meanwhile, the resulting nanocomposites maintained a high zeta potential, which is critical for colloidal stability.

### 3.2. Effect of Composition Ratio on the Performance of Nanocomposites

#### 3.2.1. Turbidity, Particle Size, PDI, and Zeta Potential

The composition ratio is a key factor influencing the formation and properties of nanocomposites, as it determines charge balance during the complexation process [32,33]. In particular, SH exhibits complex self-assembly behavior at pH values below 6, making it essential to investigate how different SH:CS mass ratios affect the characteristics of the nanocomposite.

**Figure 2:**
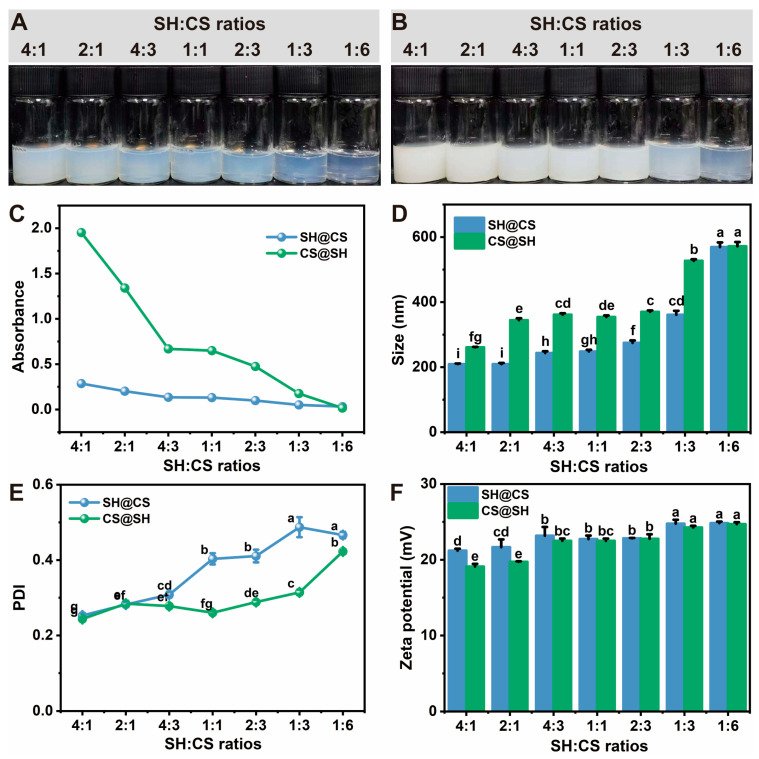
Appearance of (**A**) SH@CS and (**B**) CS@SH nanocomposites at different SH:CS ratios. (**C**) Turbidity, (**D**) particle size, (**E**) PDI, and (**F**) zeta potential of nanocomposites at different SH:CS ratios. Data in panels D-F are presented as mean ± standard deviation (*n* = 3). Different letters denote statistically significant differences among ratios (*p* < 0.05).

As shown in Figure 2A,B, the dispersion gradually transitioned from turbid to transparent as the SH:CS ratio decreased. To quantify this observation, turbidity was measured for all samples (Figure 2C). The results confirmed that even at the same total solute concentration, dispersions with a higher proportion of SH were significantly more turbid. Although the total solute content was constant, the actual mass of solutes assembled into solid nanoparticles could not be quantified and likely varied with the SH:CS ratio. Therefore, the increased turbidity observed at higher SH proportions likely results from two concurrent factors: 1) the self-assembly of a greater number of SH nanoparticles and 2) the simultaneous adsorption of more CS molecules onto these newly formed nanoparticle surfaces. Regardless of the ratio, CS@SH samples consistently appeared more turbid than SH@CS samples. This difference likely arises from variations in SH self-assembly and its interaction with CS between the two preparation routes. In the SH@CS method, a portion of SH underwent localized self-assembly into nanoparticles, which were subsequently coated by CS via electrostatic attraction. In contrast, in the CS@SH approach, electrostatic complexation occurred initially between CS and SH monomers, followed by hydrophobically driven organization. This latter pathway may produce larger or more aggregated nanocomposite structures, resulting in higher turbidity.

As shown in Figure 2D, CS@SH nanocomposites generally exhibited larger hydrodynamic sizes than their SH@CS counterparts, except at an SH:CS ratio of 1:6. These size differences likely stem from distinct assembly mechanisms dictated by the order of component addition. It should be noted that dynamic light scattering measures the overall hydrodynamic size and cannot resolve the internal architecture of nanocomposites; thus, the proposed mechanisms represent plausible interpretations consistent with our data. For SH@CS, SH nanoparticles likely formed first and were then coated by CS, a process that restricted further aggregation and growth. Conversely, in CS@SH, early electrostatic interactions between CS and SH monomers might have led directly to the formation of larger coacervates or aggregates. This mechanistic framework helps explain the observed trends. In the SH@CS system, particle size increased as the SH:CS ratio decreased. A higher SH content produced compact SH nanoparticles that promoted stronger electrostatic interactions with CS, yielding smaller nanocomposites. As the SH proportion decreased, electrostatic attraction weakened, producing larger SH@CS particles—particularly at ratios of 1:3 and 1:6—suggesting that excess CS was adsorbed onto SH nanoparticle surfaces. For CS@SH, increasing the CS ratio also resulted in larger particles; however, there was little variation in size between SH:CS ratios of 2:1 and 2:3, indicating optimal complexation in this range. At low SH levels, although electrostatic interactions remained strong, the limited SH content could not fully balance the excess CS, hindering the formation of compact nanocomposites.

As shown in Figure 2E, all CS@SH nanocomposites exhibited PDI values below 0.3 (except at the 1:6 SH:CS ratio), indicating good dispersibility. In contrast, SH@CS nanocomposites maintained PDI values below 0.3 only at SH:CS ratios of 4:1 and 2:1. This difference may result from the relatively loose and heterogeneous adsorption of CS on SH nanoparticle surfaces, which reduced particle uniformity. Zeta potential is a critical parameter for evaluating colloidal stability. As shown in Figure 2F, zeta potential was the lowest at high SH:CS ratios (4:1 and 2:1) and increased markedly as the SH:CS ratio decreased (4:3, 1:1, and 2:3). The highest value, approximately +25 mV, was observed at SH:CS ratios of 1:3 and 1:6. This trend suggests that CS predominantly determined the zeta potential of the nanocomposites, and increasing CS content improved colloidal stability. Notably, at SH:CS ratios of 4:1 and 2:1, SH@CS nanocomposites displayed significantly higher zeta potential than CS@SH nanocomposites, whereas no significant differences were observed between the two configurations at lower SH:CS ratios. This discrepancy may arise from different assembly mechanisms. At low SH:CS ratios, SH@CS nanocomposites likely formed through the initial assembly of SH nanoparticles followed by CS coating, whereas CS@SH nanocomposites formed via co-assembly, during which electrostatic interactions consumed more of the surface-positive charges of CS. As the CS proportion further increased, SH tended to become embedded within the nanocomposite structure, while CS was distributed on the surface. Consequently, the measured zeta potential primarily reflected the surface properties of CS, resulting in minimal differences between the two assembly routes.

#### 3.2.2. Sedimentation Tendency of Nanocomposites After Storage

The DF after storage for 15 days at 4 °C serves as a key indicator of the colloidal stability of the nanocomposites, reflecting their resistance to aggregation and sedimentation over time. For pure SH, protonation of carboxylate groups under acidic conditions promoted self-assembly into nanoparticles. However, as the surface charge weakened over time, electrostatic repulsion decreased, leading to aggregation and eventual precipitation. As shown in Table 1, nanocomposites with higher SH content exhibited lower DF values. Although the nanocomposites carried positive charges, they still tend to aggregate and precipitate. With increasing CS content, the DF progressively improved and eventually reached 100%, indicating enhanced stability. This improvement is attributed to the dual stabilizing effects of the excess CS chains adsorbed on nanoparticle surfaces. First, the protruding CS chains created a steric barrier that physically prevented close particle contact. Second, as a polycation, CS increased the surface positive charge, enhancing electrostatic repulsion between nanocomposites. The combination of steric and electrostatic stabilization effectively suppressed aggregation and sedimentation.

At SH:CS ratios above 2:3, SH@CS nanocomposites exhibited lower DF values than their CS@SH counterparts. This difference can be attributed to their distinct formation pathways. In the SH@CS method, SH first self-assembled into nanoparticles in the acidic CS solution. When the amount of CS was insufficient to coat and stabilize these pre-formed SH cores fully, aggregation could not be effectively suppressed. In contrast, in the CS@SH method, SH directly interacted with CS through stronger electrostatic attraction during pH adjustment, leading to the formation of nanocomposites with inherently greater colloidal stability and improved resistance to sedimentation.

### 3.3. Transmission Electron Microscopy Analysis

**Figure 3:**
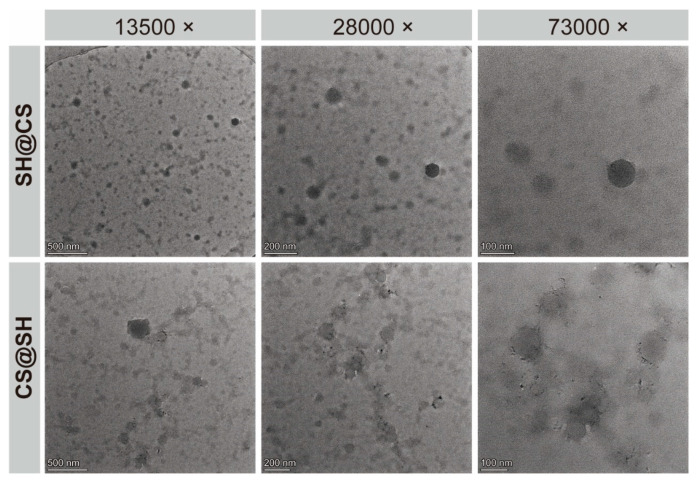
TEM images of SH@CS and CS@SH nanocomposites at an SH:CS ratio of 2:3.

To examine the morphology of the SH-CS nanocomposites, TEM images of samples prepared at an SH:CS mass ratio of 2:3 were obtained at multiple magnifications. As shown in Figure 3, both SH@CS and CS@SH exhibited particulate morphologies, but with marked differences. SH@CS nanocomposites appeared well-dispersed and predominantly spherical, whereas CS@SH nanocomposites were mostly irregular and exhibited noticeable aggregation and adhesion. In addition, SH@CS nanocomposites were substantially smaller than CS@SH nanocomposites, consistent with the hydrodynamic size trend presented in Figure 2D. Interestingly, this difference in particle size and aggregation state cannot be explained solely by the final surface charge, as both systems showed similarly high positive zeta potentials (approximately +23 mV; Figure 2F). Typically, a high zeta potential confers strong electrostatic repulsion that prevents aggregation. The fact that CS@SH nanocomposites were larger despite this strong repulsion suggests that their size was determined primarily during the initial rapid formation step, rather than by subsequent aggregation. These morphological differences likely result from the distinct formation mechanisms governed by the order of component addition. We hypothesized that, in the SH@CS route, the rapid pH decreases induced SH to self-assemble into nanoparticle cores, which subsequently interacted with surrounding CS chains. The excess CS in the continuous phase provided electrostatic and steric stabilization, limiting further particle–particle adhesion. In contrast, in the CS@SH route, initial electrostatic complexation between SH and CS, followed by hydrophobically driven compaction, favored the formation of larger and more irregular aggregates. It should be noted that particle sizes observed by TEM were expectedly smaller than the hydrodynamic diameters measured by dynamic light scattering (Figure 2D). This well-known discrepancy arises because dynamic light scattering measured the fully hydrated particle size in solution, which included the solvated polymer corona, whereas the dehydration process during TEM sample preparation inevitably caused particle shrinkage [34].

### 3.4. Fourier Transform Infrared Spectroscopy Analysis

**Figure 4:**
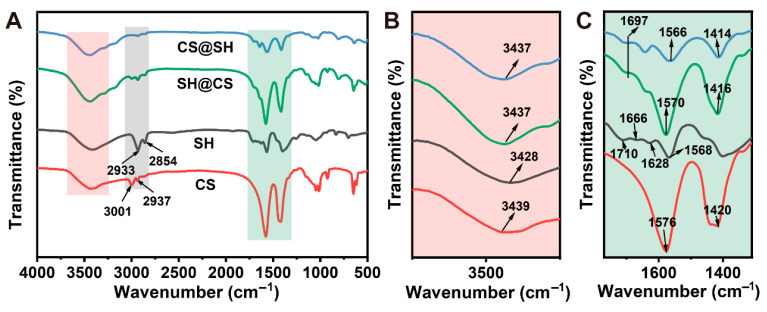
(**A**) FTIR spectra of SH, CS, and their nanocomposites; (**B**) enlarged view of the region from 3250 to 3750 cm^−1^; and (**C**) enlarged view of the region from 1310 to 1770 cm^−1^.

FTIR was used to evaluate molecular structural changes and intermolecular interactions. The spectra of CS, SH, and their nanocomposites are presented in Figure 4. The SH spectrum exhibited distinct absorption bands at 3428, 2933, and 2854 cm^−1^, as well as at 1710, 1666 and 1628, and 1568 cm^−1^. These bands corresponded to O-H stretching vibration of hydroxyl groups [35], C-H stretching in methylene and methyl groups, C=O stretching in esters or carboxylic acids [36], conjugated C=O or C=C vibrations, and asymmetric stretching of carboxylates [35,37], respectively. For CS, the broad band at 3439 cm^−1^ was attributed to O-H (hydroxyl) and N-H (amino) stretching vibrations, while the bands at 3001 and 2937 cm^−1^ corresponded to asymmetric and symmetric C-H stretching in methyl or methylene groups. The band at 1576 cm^−1^ was assigned to N-H bending (amino) and C=O bending (amide II), and the band at 1420 cm^−1^ corresponded to C-H bending or in-plane O-H bending [38].

For the SH@CS and CS@SH nanocomposites, the spectra retained all the characteristic absorption bands of both SH and CS. Importantly, no new peaks appeared, providing strong evidence that the complexation occurred through non-covalent interactions rather than the formation of new covalent bonds. However, notable shifts in specific bands were observed, indicating significant intermolecular interactions between the two components [39]. For instance, the broad O–H/N–H stretching bands (3400–3500 cm^−1^) and the C=O stretching band of SH at 1710 cm^−1^ both shifted to lower wavenumbers in the nanocomposites. These red shifts are characteristic indicators of hydrogen bond formation between the hydroxyl/carboxyl groups of SH and the hydroxyl/amino groups of CS [40]. Furthermore, shifts in the N–H bending peak of CS and the carboxylate peak of SH are consistent with electrostatic interactions between the positively charged amino groups of CS (–NH_3_^+^) and the negatively charged carboxylate groups of SH (–COO^−^) [41]. Subtle differences in the magnitude of these band shifts between the SH@CS and CS@SH spectra likely result from the fact that two assembly routes produced structurally distinct nanocomposites, leading to different local chemical environments for the functional groups.

### 3.5. Molecular Interaction Analysis

**Figure 5:**
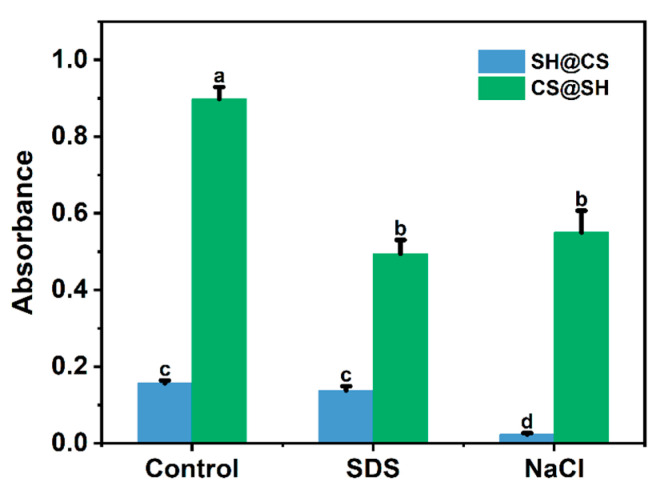
Changes in absorbance of the nanocomposites after the addition of SDS and NaCl. Data are presented as mean ± standard deviation (*n* = 3). Different letters indicate statistically significant differences among samples (*p* < 0.05).

The formation and stability of the nanocomposites are governed by multiple molecular interactions, which can be selectively disrupted by specific dissociating agents. Generally, NaCl induces electrostatic shielding, thereby weakening electrostatic interactions and destabilizing the nanocomposite structure, whereas the anionic surfactant SDS disrupts hydrophobic interactions [42,43]. As shown in Figure 5, the addition of both NaCl and SDS to CS@SH nanocomposite dispersions markedly reduced turbidity, indicating that both electrostatic and hydrophobic interactions contribute to SH-CS binding and overall nanocomposite stability. In contrast, for SH@CS nanocomposites, NaCl addition significantly decreased turbidity, while SDS caused almost no change. This seemingly contradictory observation can be explained by the proposed structural model, in which the dominant interactions were spatially segregated. In the SH@CS system, hydrophobic interactions primarily drove the initial self-assembly of SH into nanoparticle cores. The overall colloidal stability of the final nanocomposite was mainly governed by electrostatic interactions between these pre-formed cores and the stabilizing CS outer layer. Conversely, in the CS@SH system, SH interacted directly with CS to form a hybrid nanocomposite through the combined effects of electrostatic and hydrophobic interactions, so both NaCl and SDS could effectively induce dissociation. FTIR analysis further revealed that hydrogen bonding also played an important role in nanocomposite formation and stability. Urea, a common hydrogen-bond-disrupting agent, can lead to composite dissociation and turbidity reduction. However, in this system, urea can disrupt intrinsic hydrogen bonds between CS and SH by forming new hydrogen bonds with them but cannot prevent rapid SH aggregation, which severely interferes with turbidity measurements. Therefore, dissociation experiments were not suitable for directly verifying hydrogen bonding interactions in these nanocomposites.

### 3.6. Potential Mechanism of Nanocomposite Formation

**Figure 6:**
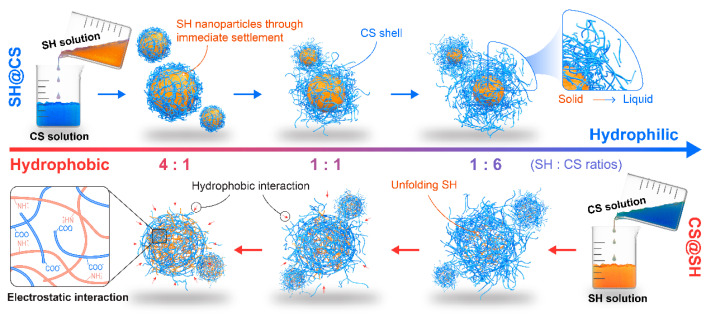
Proposed formation mechanism of SH@CS and CS@SH nanocomposites.

The pH-driven method is widely employed to construct natural polymer-based nanocomposites, as it utilizes strong electrostatic interactions between oppositely charged polymers at specific pH values. In this study, this approach was applied to design SH-CS nanocomposites with enhanced interfacial activity by leveraging the substantial solubility differences between SH and CS under distinct pH conditions and the pH variation that occurred upon mixing. However, the assembly and aggregation behavior of SH under acidic conditions could be influenced by the solvent environment, which in turn may affect the formation process and properties of the resulting nanocomposites. Based on the experimental results, a possible formation mechanism was proposed (Figure 6). When CS was added to SH, the solution pH gradually decreased, causing both SH and CS to shift from their initial unfolded states. Electrostatic interactions first brought the two components into close proximity, after which the hydrophobic domains of SH drove structural rearrangement, promoting co-assembly with CS into compact composite structures. In contrast, when SH was added to CS, local over-acidification at the mixing interface induced the rapid aggregation of SH into nanoparticles, which subsequently associated with CS during continuous stirring. The absorbed CS layer prevented precipitation through electrostatic repulsion, resulting in a stable dispersion composed of SH–CS nanocomposites and free CS.

### 3.7. Interface Properties

#### 3.7.1. Three-Phase Contact Angle Analysis

The three-phase contact angle of a compressed pellet is commonly used to evaluate the wettability of powdered materials. However, it is important to recognize the inherent limitations of this technique when interpreting the results. The surface of a pressed pellet is neither perfectly smooth nor uniform; its roughness and porosity can influence the measured (apparent) contact angle. Furthermore, the static and consolidated nature of the pellet may not fully reflect the dynamic behavior of individual nanocomposites at a fluid droplet interface. Therefore, the contact angle values (θ) reported here should be regarded as a relative indicator of the collective surface wettability of the nanocomposites, rather than absolute measures corresponding directly to the behavior of individual particles in applications such as emulsion stabilization.

**Figure 7:**
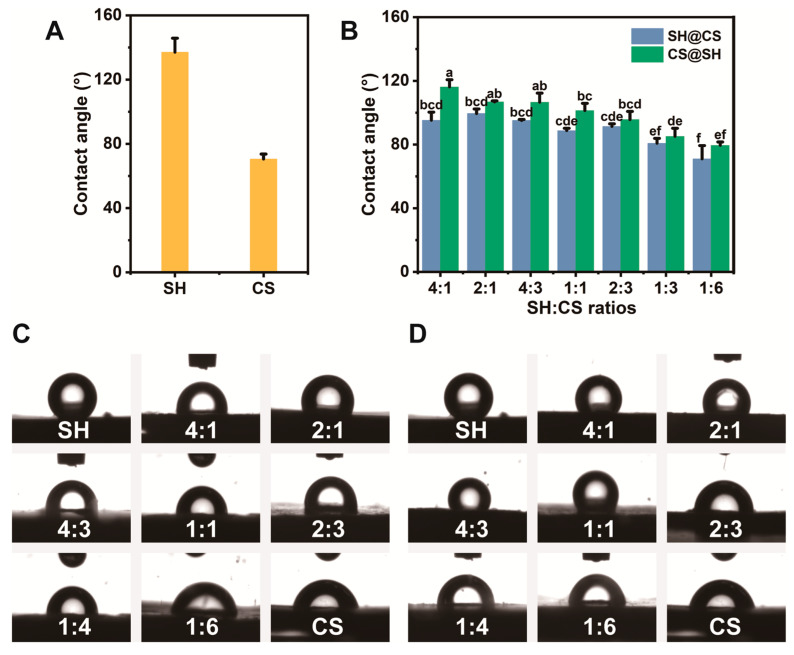
(**A**) Three-phase contact angles of SH and CS; (**B**) three-phase contact angles of SH-CS nanocomposites; (**C**) representative contact angle images of SH@CS nanocomposites; (**D**) representative contact angle images of CS@SH nanocomposites. Data are presented as mean ± standard deviation (*n* = 3). Different letters indicate statistically significant differences among different ratios (*p* < 0.05).

With these considerations in mind, the three-phase contact angle measurements revealed clear trends in surface properties. As shown in Figure 7A, a pellet of pure SH exhibited a three-phase contact angle of approximately 140°, indicating a strongly hydrophobic surface, whereas pure CS showed a three-phase contact angle of about 75°, consistent with its hydrophilic nature. When SH and CS were combined, the apparent wettability of the nanocomposites varied systematically with the CS proportion, suggesting that CS effectively modulated the inherent hydrophobicity of SH. At a mass ratio of 2:3 (SH:CS), the three-phase contact angle approached 90°, indicating a surface with balanced wettability (Figure 7B). For SH@CS nanocomposites, the three-phase contact angle was approximately 92° at a mass ratio of 4:1. Further increases in CS content caused little change until the ratio reached 1:6, where the three-phase contact angle dropped sharply to ~74°, indicating a predominantly hydrophilic surface (Figure 7C). In contrast, CS@SH nanocomposites exhibited a more consistent downward trend in three-phase contact angle with increasing CS proportion (Figure 7D). These distinct trends can be explained by their proposed structural organization. In SH@CS, SH nanoparticles formed the core, with CS coating the surface. The hydrophilicity of the pellet surface primarily depended on the extent of CS coverage. Once the SH cores were fully coated, the surface was dominated by CS, and additional CS contributed little change in wettability. In contrast, in CS@SH, incomplete coverage at low CS content left hydrophobic SH regions exposed, while higher CS proportions effectively masked SH’s hydrophobicity, leading to a progressive reduction in three-phase contact angle.

#### 3.7.2. Interfacial Tension Analysis

**Figure 8:**
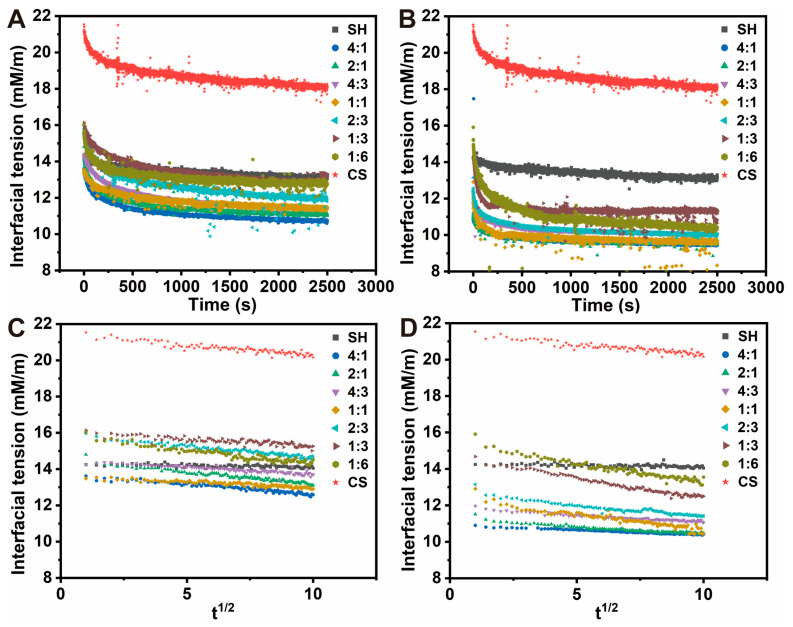
Dynamic interfacial tension at the oil-water interface for nanocomposites with various SH:CS mass ratios. (**A**) SH@CS and (**B**) CS@SH plotted against time (t); (**C**) SH@CS and (**D**) CS@SH plotted against the square root of time (t^1^ᐟ^2^) for adsorption kinetics analysis.

Interfacial tension is a key parameter governing the adsorption of nanocomposites at the oil–water interface, directly influencing emulsion formation and stability [44,45]. The temporal evolution of interfacial tension (Figure 8A,B) showed an initial rapid decrease followed by a slower decline toward a plateau, reflecting the progressive occupation of the oil–water interface by surface-active species and formation of a structured, possibly viscoelastic, interfacial layer [22,46]. Compared with SH and CS alone, the nanocomposites achieved lower equilibrium interfacial tension, indicating enhanced surface activity and a greater ability to form stable interfacial films. However, when the CS proportion exceeded the optimal range, the equilibrium interfacial tension increased, likely because elevated continuous-phase viscosity and/or excessive surface hydrophilicity hindered nanocomposite adsorption and rearrangement. Notably, CS@SH consistently exhibited lower equilibrium interfacial tension than SH@CS, suggesting that CS@SH nanocomposites possessed higher thermodynamic potential to reduce interfacial energy, a critical property for effective stabilizers.

To move beyond this equilibrium perspective and evaluate adsorption efficiency, the adsorption kinetics were analyzed using the Ward–Tordai model (Figure 8C,D). This analysis quantified the initial adsorption rates and revealed marked differences between the two systems. The SH@CS nanocomposites exhibited an adsorption rate comparable to that of pure CS, indicating that adsorption kinetics were primarily governed by the external CS layer. This observation aligned well with the proposed core–shell structure of SH@CS, in which the CS shell controlled the interaction of the particles with the oil–water interface. In contrast, the adsorption rate of CS@SH strongly depended on the SH:CS ratio and, at optimal ratios (SH:CS < 1:1), even exceeded that of either pure component. This synergistic kinetic behavior supported the formation of a co-assembled structure, where molecular entanglement between SH and CS facilitated faster and more efficient migration and arrangement at the interface.

In summary, the kinetic data directly supported the central hypothesis that the assembly sequence determined the nanocomposite structure, which in turn governed not only the thermodynamic effectiveness but also the kinetic efficiency of interfacial stabilization.

### 3.8. Emulsification Properties

**Figure 9:**
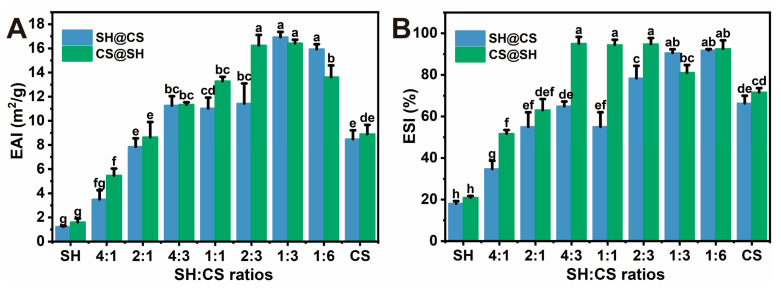
(**A**) EAI and (**B**) ESI of SH-CS nanocomposites. Data are presented as mean ± standard deviation (*n* = 3). Different letters denote statistically significant differences among different ratios (*p* < 0.05).

The EAI and ESI, determined by turbidimetric analysis, are commonly used to evaluate the initial emulsifying ability of nanoparticles and the short-term stability of the emulsions against coalescence [29,30]. As shown in Figure 9, pure SH exhibited poor emulsification performance due to its strong hydrophobicity, while pure CS also showed weak emulsification because of its pronounced hydrophilicity [12,19]. A high SH proportion markedly reduced both EAI and ESI; however, incorporating CS into SH effectively mitigated this limitation. This improvement can be attributed to the formation of nanocomposites, which minimized SH aggregation and provided a more balanced interfacial wettability. Similar enhancements in interfacial wettability have been reported in composite particles composed of zein and CS [21]. The highest ESI value, indicating the greatest resistance to coalescence within the measurement period, was observed at an SH:CS mass ratio of 2:3. Beyond this ratio, further increases in CS content led to a decrease in EAI, likely because excessive CS produced an overly hydrophilic surface that diminished interfacial activity [23]. Notably, the CS@SH system consistently exhibited higher EAI and ESI values than SH@CS, suggesting superior emulsifying ability and initial stabilization capacity. This difference aligns with the proposed structural models. The hypothesized hybrid co-assembled structure of CS@SH, characterized by a more uniform distribution of hydrophilic and hydrophobic groups, likely facilitated more effective packing and interfacial network formation, resulting in higher EAI and ESI. In contrast, in the core–shell structure of SH@CS, the increasingly hydrophilic surface at higher CS contents could reduce interfacial adsorption efficiency, accounting for its lower emulsification performance.

### 3.9. Potential Food Industry Applications

The findings of this study provide a practical “design rule’’ for creating tunable, food-grade Pickering stabilizers with broad potential in the food industry. The CS@SH nanocomposites, which demonstrated superior emulsifying performance, can be particularly valuable in formulating long-shelf-life emulsions, such as salad dressings, mayonnaise, and creamy sauces, where their ability to form robust interfacial films may enhance stability against processing stresses and during storage. Beyond basic stabilization, a key application lies in the encapsulation and delivery of active compounds [47,48]. For example, these nanocomposites can serve as effective carriers for essential oils, forming stable nanoemulsions with antimicrobial properties. Such emulsions can be incorporated into edible coatings or films for active food packaging to extend shelf life or introduced into food matrices, such as baked goods, to impart unique flavors and provide preservative effects. Additionally, these nanocomposites can encapsulate bioactive compounds such as curcumin, β-carotene, lutein, and n-3 polyunsaturated fatty acids, enhancing the nutritional value of functional beverages and fortified foods [49].

Furthermore, the ability of these Pickering emulsions to mimic the structure and sensory properties of fat globules opens opportunities for developing healthier food products through fat-replacement strategies [50]. This approach is particularly relevant for low-fat or low-saturated-fat products, such as ice cream and yogurt, where emulsion droplets can act as fat substitutes to maintain a creamy texture. In the emerging field of alternative proteins, these emulsions can also be engineered to replace animal fats in plant-based meat analogs, improving their juiciness and nutritional profile. Perhaps the most advanced application lies in exploiting the pH-responsive properties of SH and CS. Due to the pH-dependent solubility of both polymers, the integrity of the nanocomposite layer can be modulated during passage through the gastrointestinal tract. This provides the opportunity to design ‘’smart’’ emulsions that control the rate and extent of lipid digestion, which may promote satiety or enable the development of healthier food options. Similarly, this pH-responsive behavior can be harnessed to modulate the bioavailability of encapsulated bioactive compounds, ensuring targeted release in specific regions of the digestive system.

## 4. Conclusions

This study systematically examined how the solution addition sequence affects the formation, physicochemical properties, and interfacial activity of pH-driven SH-CS nanocomposites. We found that pH controlled the ionization state of SH and CS and that near pH 5, favorable electrostatic interactions occurred between them, promoting colloidal nanocomposite formation under the experimental conditions. The SH:CS mass ratio strongly influenced the nanocomposite properties, with a mass ratio of 2:3 yielding nanocomposites exhibiting optimal zeta potential and the highest colloidal stability. The two assembly routes produced distinct morphologies: the SH@CS method yielded nanocomposites consistent with a core–shell structure, whereas the CS@SH method produced co-assembled structures. These structural differences, driven by different balances of intermolecular forces, resulted in distinct interfacial properties and emulsifying performance. Crucially, these findings establish a practical design rule for creating effective food-grade Pickering stabilizers. To achieve superior emulsifying performance, such as higher EAI and ESI values and lower equilibrium interfacial tension, at a pH near 5, our results suggest targeting an SH:CS mass ratio of approximately 2:3 and employing the CS@SH assembly route (adding CS into SH). Future optimization could involve controlled, slow dosing of the CS solution to mitigate local pH fluctuations. Additionally, validating this design rule under controlled ionic strength conditions would be a valuable direction for future work.

## Figures and Tables

**Table 1:** The dispersed fraction of SH@CS and CS@SH nanocomposites after storage for 15 days at 4 °C.

SH:CS Ratios	The DF of SH-CS Nanocomposites (%)	
	SH@CS	CS@SH
4:1	63.06 ± 4.040 ^d^	74.41 ± 4.294 ^c^
2:1	63.06 ± 4.876 ^d^	76.48 ± 4.624 ^c^
4:3	65.62 ± 2.221 ^d^	85.33 ± 1.177 ^b^
1:1	87.24 ± 4.084 ^b^	95.65 ± 0.602 ^a^
2:3	100.00 ± 0.000 ^a^	97.54 ± 1.475 ^a^
1:3	100.00 ± 0.000 ^a^	100.00 ± 0.000 ^a^
1:6	100.00 ± 0.000 ^a^	100.00 ± 0.000 ^a^

Data are presented as mean ± standard deviation (*n* = 3). Different letters indicate statistically significant differences among ratios (*p* < 0.05).

## Data Availability

The data presented in this study are available on request from the corresponding author. The data are not publicly available due to privacy restrictions.
